# Parylene-coated platinum nanowire electrodes for biomolecular sensing applications

**DOI:** 10.3762/bjnano.16.101

**Published:** 2025-08-20

**Authors:** Chao Liu, Peker Milas, Michael G Spencer, Birol Ozturk

**Affiliations:** 1 Department of Biology, Morgan State University, Baltimore, MD 21251, USA; 2 Department of Physics & Engineering Physics, Morgan State University, Baltimore, MD 21251, USA; 3 Center for Research and Education in Microelectronics, Morgan State University, Baltimore, MD 21234, USA; 4 Department of Electrical and Computer Engineering, Morgan State University, Baltimore, MD 21234, USA; 5 Department of Electrical and Computer Engineering, Cornell University, Ithaca, NY 14850 USA

**Keywords:** biosensor, directed electrochemical nanowire assembly (DENA), dopamine, glucose, nanoelectrode, platinum

## Abstract

Nanoscale biosensors have gained attention in recent years due to their unique characteristics and size. Manufacturing steps, cost, and other shortcomings limit the widespread use and commercialization of nanoscale electrodes. In this work, a nano-size electrode fabricated by directed electrochemical nanowire assembly and parylene-C insulation is introduced. Results show that the diameter of the platinum nanowire and electrode tip length can be tuned down to 120 nm and 1.2 µm, respectively, where the exposed nanowires on the electrode tips are chemically active and their surfaces can be modified for specific biosensing applications. The biosensing testing with glucose and dopamine demonstrate limits of detection of 30 nM and 0.01 mM, respectively. The *R*-squared values for peak current versus concentration are 0.985 and 0.994, indicating strong linear correlations. These nanoscale electrodes hold great promise for single-cell biosensing applications due to their compact size, biocompatibility, and rapid fabrication.

## Introduction

Electrodes, a tool used in all walks of life today, were first demonstrated by Max Cremer in 1906 [1]. After 100 years of evolution, electrodes have become ubiquitous across numerous industries including medical diagnostics, food safety, agricultural and environmental monitoring, bioprocess management, and more [2]. Despite the differences in design and function, all electrodes share similar requirements and components [3]. Advancements in materials science over the past two decades have driven significant progress in the development of nanoscale electrodes. Their high surface-to-volume ratio and nanoscale dimensions offer inherent advantages, including improved response time, improved sensitivity, and enhanced signal-to-noise ratio besides high-resolution scanning applications [4–5]. These properties have made nanoscale electrodes the preferred choice in the biosensing industry, particularly for point-of-care applications. As this market continues to expand, there is a growing demand for more portable and reliable devices [6].

Contemporary nanoscale biosensors are engineered for diverse applications, employing a variety of materials and design architectures to optimize performance and functionality. For example, carbon nanotubes (CNTs) are categorized as either single-walled or multiwalled, with diameters ranging from 0.4 to 100 nm and lengths extending from a few micrometers to several centimeters [7]. The functionalization of CNTs is achieved by surface modification through various methods including covalent bonding, adsorption, and detection group attachment [7–8]. Palve et al. developed a glucose sensor using copper nanowires and CNTs, achieving a limit of detection as low as 0.3 nM, highlighting the remarkable sensitivity of CNT-based electrodes [9]. Nevertheless, several studies reported the toxicity of CNTs for tissues and cells including loss of cellular integrity, DNA damage, and cell oxidative stress [10]. Another important class of biosensors relies on metal nanoparticles, as metals have long served as some of the earliest and most widely utilized materials in biosensor development. Like CNTs, nanoscale metal particles benefit from their small size and high surface-to-volume ratio, enhancing sensitivity and functionalization capabilities. Baetsen-Young et al. used dextrin-capped gold nanoparticles (12 nm) with ssDNA to detect the presence of *Pseudoperonospora cubensis* DNA, where the results show the ability to detect 18 spores/µL crude extractions [11]. However, the stability of metal nanoparticles limits their application in biological systems and decreases their performance [12]. Other materials like silicon quantum dots, ion-imprinted polymer, and hydrogels have unique biocompatibility and specificity advantages, but long-term stability and cost make them inappropriate for some applications [13–15].

Among various manufacturing techniques, directed electrochemical nanowire assembly (DENA) stands out as a one-step method that enables rapid and tunable fabrication of crystalline metal nanowires [16–17]. In the DENA method, AC power and a metal salt (e.g., Au, Pt, Fe, Cu, or Ir) solution are used for nanowire growth, where adjustments to the AC field parameters and solution composition allow the fabrication of either single cylindrical nanowires (ranging from 15 to 600 nm in diameter and up to several hundreds of micrometers in length) or dendritic structures [18–21]. These characteristics allow DENA to create a variety of structures for nanoscale biosensor manufacturing. Parylene-C is a polymer known for its exceptional properties, including high tensile strength, excellent electrical insulation, low water permeability, and biocompatibility [22]. Due to these attributes, parylene-C, along with other variants of parylene, has been extensively utilized in the protection of electronic devices and surface modification of biomedical devices [23].

In this study, platinum was chosen as the DENA growth material due to its exceptional biocompatibility, catalytic efficiency, and stability [24]. Parylene-C was utilized for the encapsulation and insulation of the electrodes. Dopamine and glucose were selected as analytes for testing biosensing capabilities of the electrodes. Nanoscale electrode growth steps are explained including nanowire growth, parylene-C encapsulation, and laser-assisted nanowire tip exposure. The nanowire electrode structure and the corresponding functionality are described. Electrochemical testing results of dopamine and glucose detection are also presented.

## Results and Discussion

**Figure 1:**
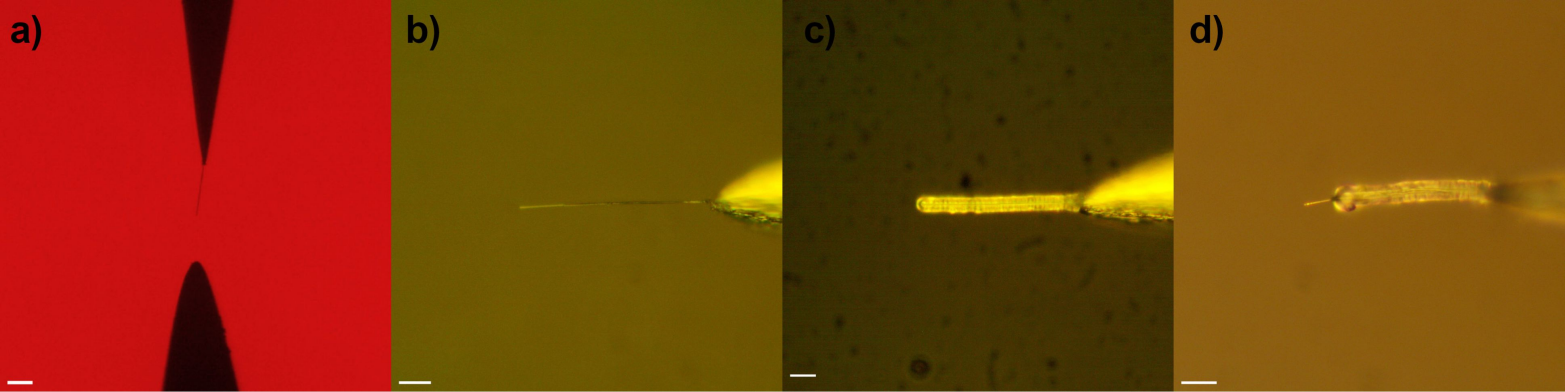
Optical images of (a) a DENA-grown platinum nanowire between two electrodes while still in the growth solution, (b) a platinum nanowire in air after removed from the growth solution, (c) a single platinum nanowire coated with parylene-C polymer, and (d) a laser-exposed platinum electrode. Scale bars are 10 μm.

After optimization of the growth parameters such as AC voltage frequency and solution concentration (see Methods section), the resulting DENA grown platinum nanowires had diameters ranging from 120 to 600 nm and lengths varying from a few micrometers to over 100 micrometers. The thickness of the parylene-C coating can be adjusted from 0.6 to 15 µm by controlling both the coating time and parylene-C vapor pressure. The thickness of the parylene-C coating was proportional to the product of pressure and time (Pa × min). As Figure 1 shows, a straight cylindrical platinum nanowire can be obtained with the custom-built DENA nanowire growth setup, which can be coated with parylene-C polymer and the tip can be exposed by a focused laser beam.

**Figure 2:**
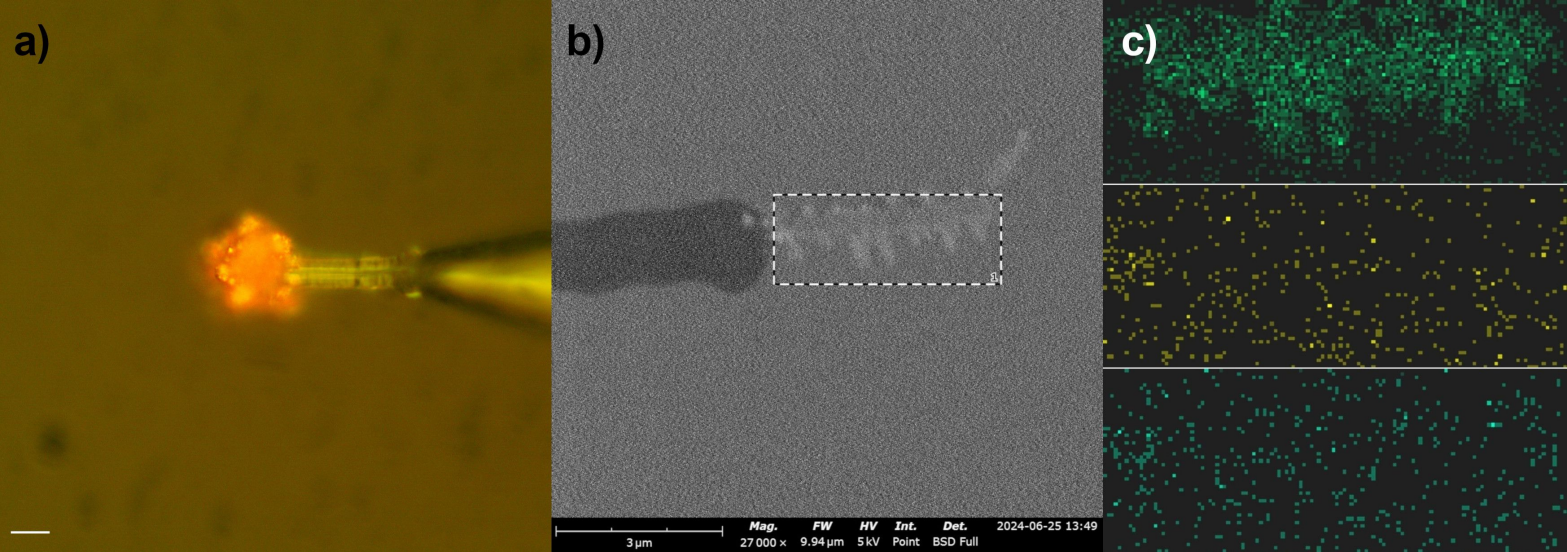
(a) Optical image of electrochemically deposited copper nodule on platinum nanowire tip. Scale bar is 10 μm. (b) SEM image of the deposited thin copper layer on the platinum nanowire tip of an electrode. (c) EDS elemental characterization of the copper deposition in panel b (from top to down: copper, platinum, and carbon).

An electrochemical copper deposition test was performed to verify that the platinum electrode was fully exposed after evaporation of the parylene-C polymer at the tip. The test also demonstrated that the remaining parylene-C polymer on the rest of the electrode acted as an insulator. As shown in Figure 2a, a copper nodule was observed on the tip of the nanowire. Copper deposition on the tungsten metal part after parylene deposition and laser evaporation indicated that the parylene insulation was not complete as intended and these electrodes were discarded. A thin layer of copper deposition on the exposed platinum nanowire tip was further examined using SEM imaging and energy-dispersive X-ray spectroscopy (EDS) elemental composition analysis. Figure 2b presents an SEM image of the copper-coated platinum nanowire. EDS analysis of the selected region (dashed rectangle) in Figure 2b reveals that it is predominantly composed of copper dendrites (Figure 2c), only a small part of the platinum nanowire near parylene-C was not coated.

**Scheme 1:**
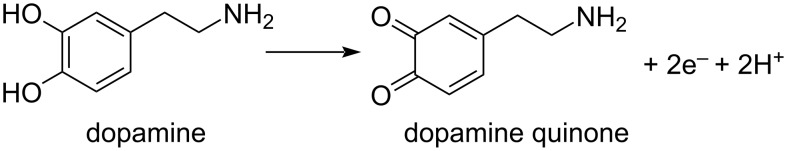
Oxidation reaction of dopamine.

The electrodes with exposed platinum nanowire tips were tested in the detection of dopamine and glucose with differential potential voltammetry (DPV). The characteristic oxidation peak for dopamine is around 150 mV, where dopamine loses two electrons and hydrons to form dopamine quinone as shown in Scheme 1 [25].

**Figure 3:**
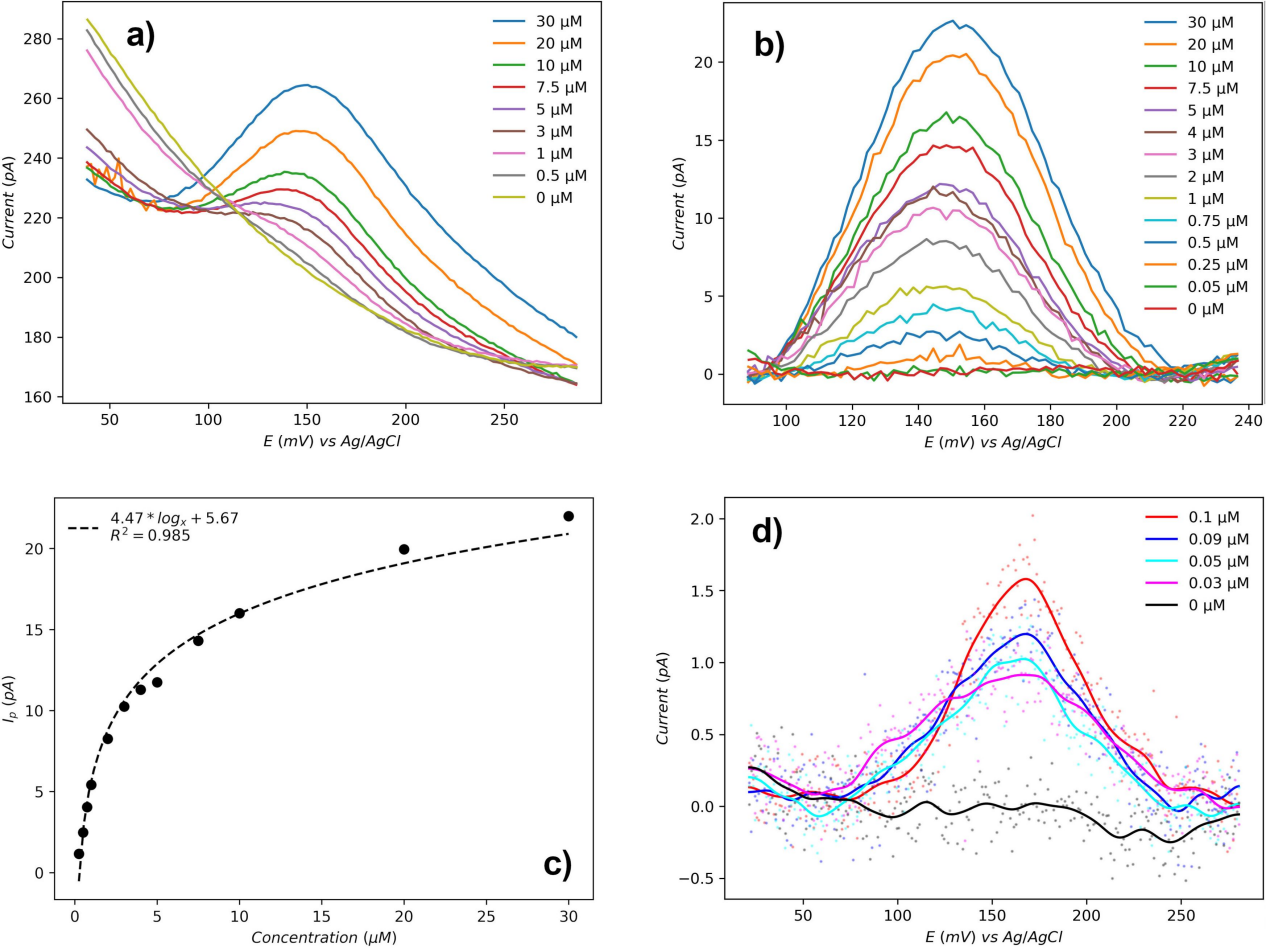
(a) Selected differential potential voltammetry (DPV) curves for dopamine detection. (b) Peak current analysis after baseline subtraction for logarithmic fitting. (c) Logarithmic fit for peak current as a function of dopamine concentration. (d) LoD analysis results at low concentrations after baseline subtraction.

For a clear view, only a few exemplary curves were used to show the DPV results in Figure 3a. Dopamine concentration in human urine is 0.2 to 3.6 μM [26], thus a maximum of 30 μM dopamine solution was tested in our experiments. Baseline subtraction was performed as described in the Methods section to determine peak currents for each dopamine concentration from 0.25 to 30 μM (Figure 3b). Peak current dependence on the dopamine concentration is depicted by the logarithmic fit in Figure 3c, which may indicate the dopamine detection is an adsorption-controlled system, considering the surface area of our electrode, the concentration of dopamine, and the slow scan rate [27]. The results show that the (limit of detection) LoD for dopamine with the DENA nanoscale electrode is 30 nM as depicted in Figure 3d.

**Figure 4:**
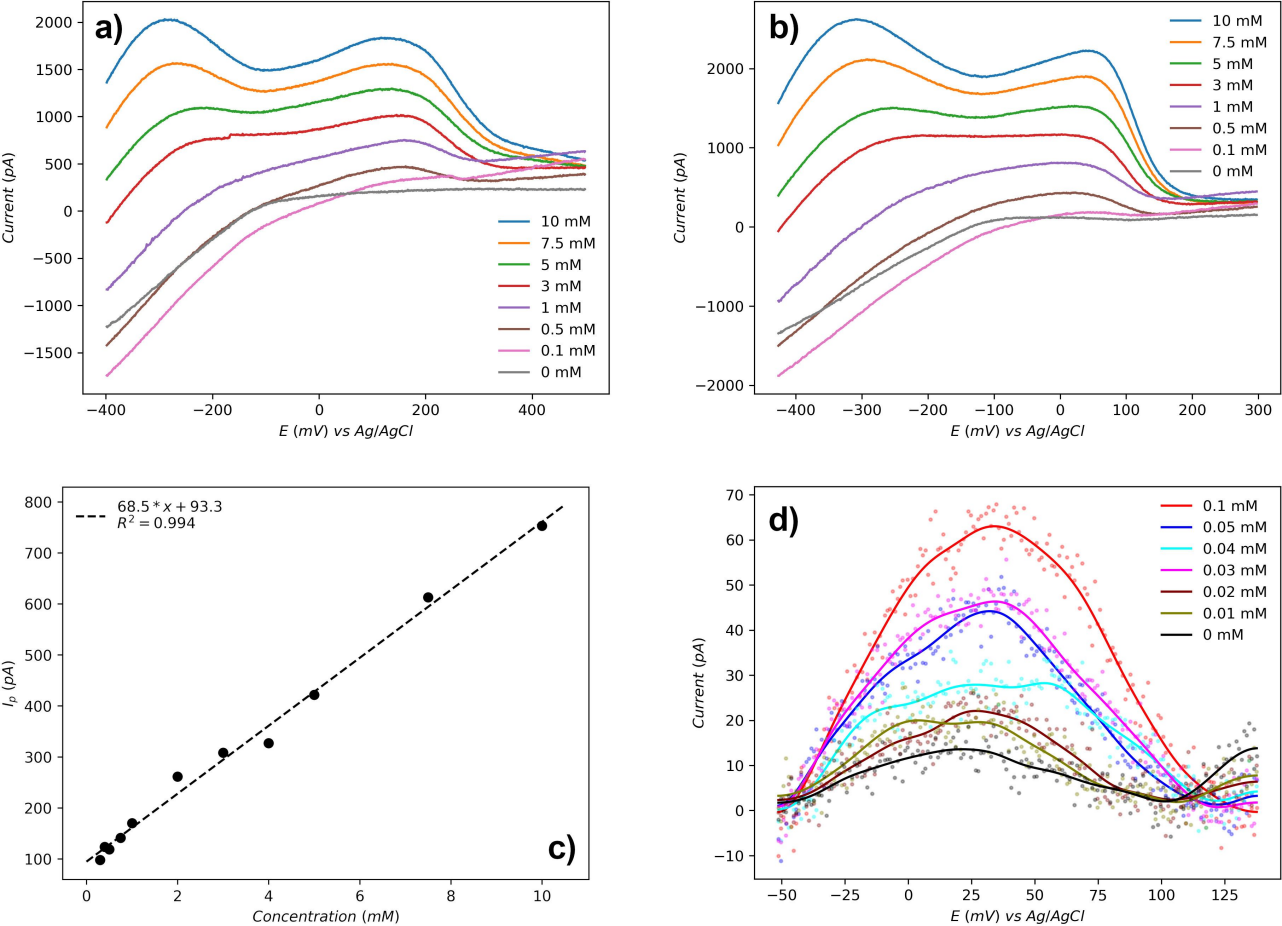
(a) Forward scan of selected glucose CV test. (b) Reverse scan of selected glucose CV test. (c) Linear fit for peak current as a function of glucose concentration. (d) LoD analysis results at low concentrations after baseline subtraction.

**Scheme 2:**
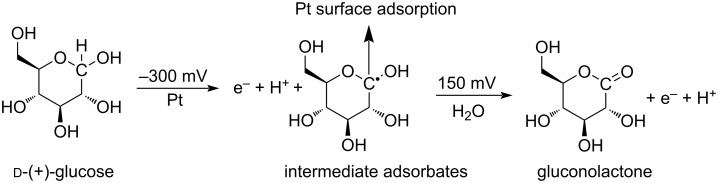
Glucose oxidation series reactions.

In glucose detection testing, two peaks were observed from both forward (Figure 4a) and reverse (Figure 4b) cyclic voltammetry (CV) scans. During forward scan, the first peak is around −300 mV, corresponding to the chemisorption onto the platinum surface and dehydrogenation of glucose. This indicates formation of intermediate adsorbate forms on the platinum surface. The second peak around 150 mV is due to the further oxidation of intermediate adsorbates with water as a catalyst, then the final product gluconolactone was generated [28–29]. These reactions were illustrated in Scheme 2. Similarly, peaks were observed to be same during the reverse scan [29]. Based on curve shape and reaction mechanism, the peaks from the reverse curve around 50 mV were selected for peak current calculation. Considering the normal range of glucose concentration in human blood ranges from 3.5 to 5.5 mM, the maximum glucose concentration was set to be 10 mM. Cyclic voltammetry testing shows a linear relationship between glucose concentration and peak current, ranging from 0.3–10 mM (Figure 4c), and the LoD is 0.01 mM glucose (Figure 4d).

With limited surface area, LoD and detection range of our nanowire electrodes are inherently constrained. However, surface modification has been shown to enhance electrode performance, as described in the Introduction section. Additionally, surface modification can improve the stability and durability of the electrode, further enhancing its applicability.

## Conclusion

This study presents a method for fabricating nano-scale electrodes with platinum nanowire tip lengths as small as 1.2 µm, and diameters down to 120 nm. This size advantage makes them suitable for biosensing applications with high spatial resolution involving single cells. The morphology and functionality of the electrodes were characterized through elemental analysis and copper deposition, demonstrating their capability for effective surface modification. Results from dopamine detection showed 30 nM LoD and a logarithmic relationship between peak current and concentration with 0.985 *R*-square score in the 0.25–30 µM concentration range. For glucose detection, the LoD was 0.01 mM, and a linear relationship between peak current and concentration with 0.994 *R*-square in the 0.3–10 mM concentration range was observed. Building on these results and prior studies, these novel nanowire electrodes hold strong potential for use as highly effective biological sensors.

### Methods

#### Tungsten rod tapering

The tungsten rod (0.254 mm (0.01 inch) diameter, A-M SYSTEMS) was etched in 2 M NaOH (98%, Fisher Scientific) solution by a modified sewing machine and a DC power supply (MPJA 9616PS). The tungsten rod was secured to the needle holder of the sewing machine and moved vertically in and out of the NaOH solution at a frequency around 5 Hz by operating the machine. The tip of the tungsten rod was immersed in the NaOH solution while applying 20 V DC to the rod and a counter electrode was also present in the NaOH solution [30]. The immersion depth of the tungsten rod in the solution was adjusted until an etching current of 0.15 A is achieved. Etching continued until the current decreased to 0.08 A. The procedure was repeated with the same parameters one more time. Subsequently, the tip of the tungsten rod was tapered to a radius of approximately 5 µm for nanowire growth.

#### DENA platinum nanowire growth

**Figure 5:**
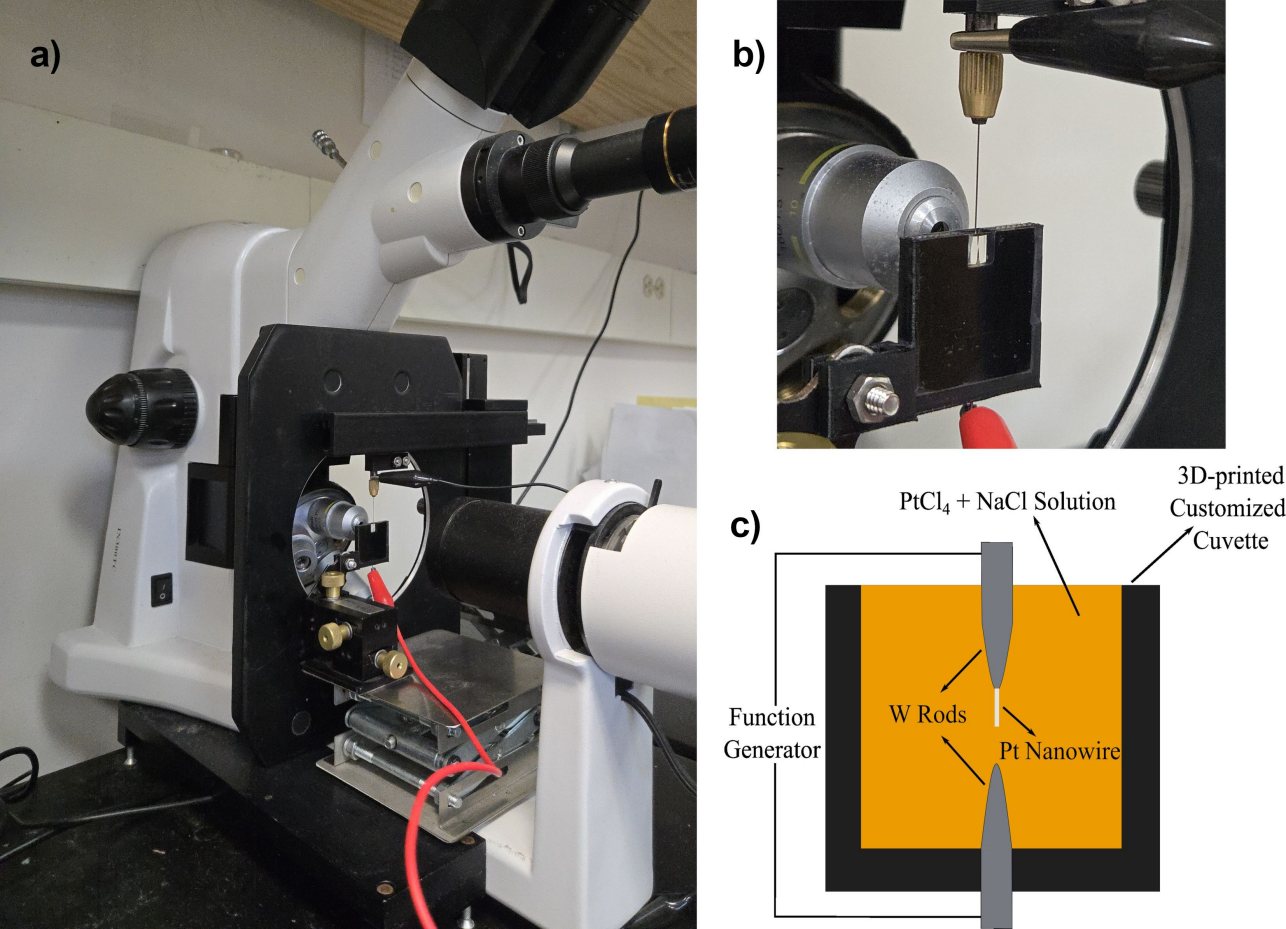
(a) DENA setup. (b) Customized cuvette. (c) Diagram of DENA setup.

Platinum nanowires were grown with the DENA method. As shown in Figure 5a, an upright microscope was tilted 90° and placed on its side for vertical nanowire growth. Horizontal nanowire growth with the DENA method results in an angle between the nanowire and the tungsten rod, which is not ideal for this nanoscale electrode application. Figure 5b shows a close-up image of the custom-built cuvette that was constructed with a 3D-printed PLA frame and two 18 mm × 18 mm cover glass pieces placed on two sides. A pair of tapered tungsten rods were immersed in the 75 mM platinum(IV) chloride (ALDRICH 96%) and 100 mM NaCl solution as depicted in Figure 5c; the addition of NaCl suppresses the growth of side branches and dendrites [31–32]. The tungsten rod at the bottom was connected to the positive output and the top rod was connected to the negative output of the function generator (BK Precision 4065). Square wave signals were applied between the two tungsten rods, where the nanowire growth occurs due to reduction of platinum ions from the solution. Frequency and peak-to-peak voltage parameters were optimized to grow a single platinum nanowire (needle-like) as opposed to dendritic nanowire branches or multiple nanowires. Starting values for growth frequency and peak-to-peak voltages were 11 MHz and 9.5 V (−4.75 V to +4.75 V), respectively, for single nanowire growth. During the growth process, the electrode separation was adjusted. Initially, the separation was reduced to a few micrometers to initiate nanowire growth. As the platinum nanowire started growing, the separation was gradually increased to 20–75 µm to prevent contact between the growing nanowire and the counter electrode, which leads to a short circuit and damages the nanowire.

#### Parylene-C coating

**Figure 6:**
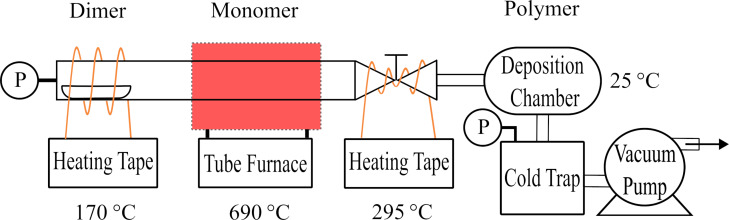
Diagram of parylene-C coating system.

The parylene-C coating system was a modified version of the setup from Gluschke and colleagues [33]. As shown in Figure 6, two BriskHeat heating tapes were used for the sublimation part (170 °C) and the valve (295 °C). A Thermcraft tube furnace was used for the pyrolysis part (690 °C), and the polymerization (deposition) chamber was an Edwards FL20K foreline trap. A BVV cold trap was used to catch uncoated parylene-C monomer, where an Edwards E2M1.5 mechanical vacuum pump was used to maintain the vacuum in the 0.133 to 1.33 Pa range during the process. A CPS VG200 vacuum gauge was used for monitoring the pressure.

Usually, 4–6 DENA-grown platinum nanowires on the tips of tungsten rods were placed 45° (from vertical) in this deposition chamber with Kapton tape. 0.5 g of parylene-C powder was placed in a ceramic boat (7 cm × 1.5 cm × 1 cm), which was used in the deposition process. The system was first evacuated to a pressure below 1.33 Pa. Next, the valve heating tape and tube furnace were activated. Once the furnace reached 690 °C (which took approximately 10 min), the sublimation heating tape was turned on. It typically took around 5 min for the sublimation to stabilize. The pressure was monitored to estimate the thickness, and the pyrolysis time was adjusted based on the pressure and the desired coating thickness. 133 Pa·min resulted in approximately 3 µm parylene-C thickness on the platinum nanowires.

After the coating process was complete, both the tube furnace and the sublimation heating tape were turned off. The valve heating tape was deactivated once the pressure returned to baseline. The vacuum pump was turned off and the deposition chamber was opened after the system had cooled down to room temperature.

#### Laser-assisted nanowire tip exposure

**Figure 7:**
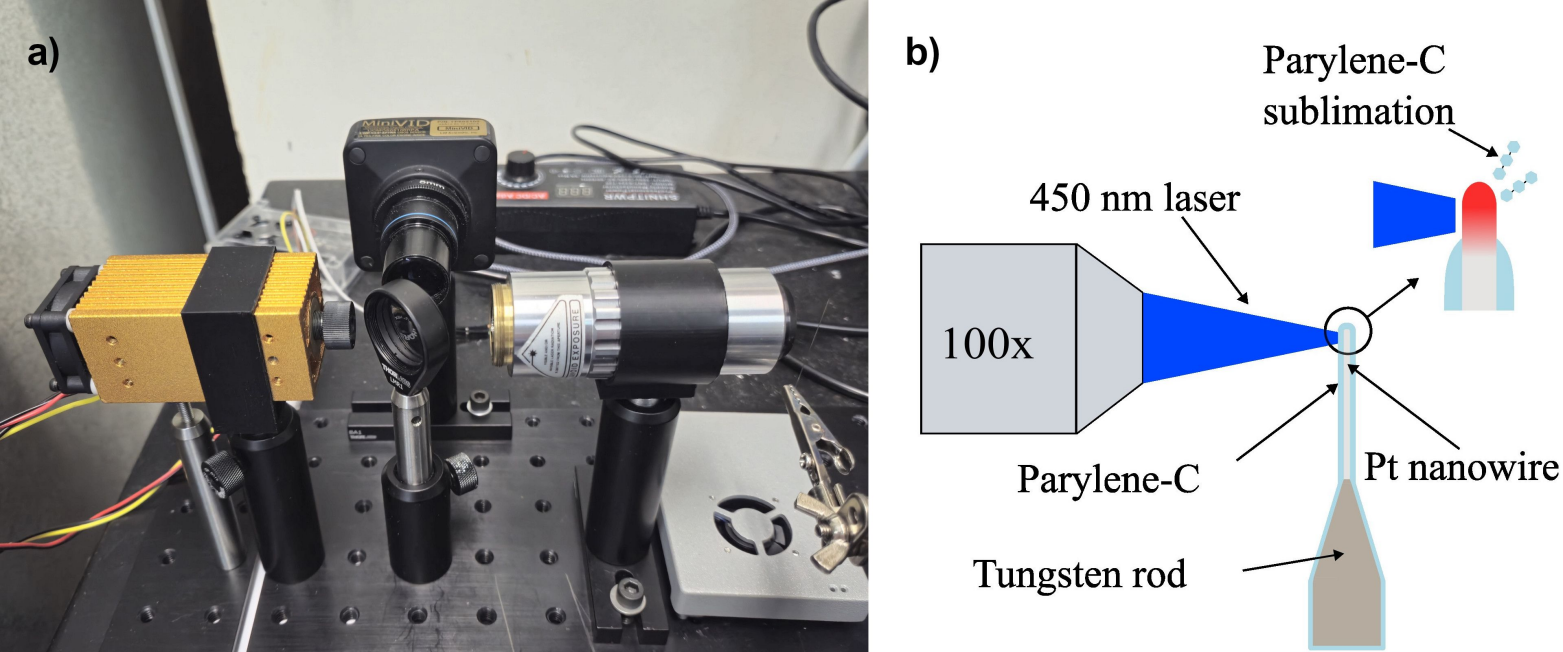
(a) Laser exposure setup. (b) Diagram of setup.

A focused 450 nm blue diode laser beam was used to expose the tip of the platinum nanowires after parylene-C coating. Figure 7 illustrates the experimental setup (Figure 7a) and a diagram of the exposure process (Figure 7b). The laser beam spot size was reduced to 35 µm diameter by a 100× long working distance objective lens (Boli Optics MT02513831). A 30R/70T beam splitter and a MU800 camera were used to place the focused laser beam spot on the tip of the nanowire electrode, where the electrode was mounted on a three-axis manual micropositioning stage. Open-source Universal G-code Sender (UGS) software was used to control laser power. The maximum power (2.85 W) of the laser was used 1 to 4 times (0.5 s each time) to sublimate the parylene-C coating. A small fan was used to blow the sublimed polymer upward to avoid deposition on the objective lens. Desired lengths of the nanowire can be exposed by adjusting the position of the electrode.

#### Copper deposition

An established method from [34] was modified to test the exposed electrode. A platinum rod was used for the counter electrode and the reference electrode is a CHI111 Ag/AgCl (3.5 M KCl) electrode. The solution was a mixture of 0.2 M CuSO_4_ and 0.1 M H_2_SO_4_. The scan window was −150 mV to 650 mV and the scan rate was 50 mV/s with three loops. In another test, concentrations were reduced to 0.02 M and 0.01 M for CuSO_4_ and H_2_SO_4_, respectively, and the scan rate was reduced to 25 mV/s for slower copper deposition. The CV test was accomplished by a DStat potentiostat [30,35].

#### Dopamine detection by differential potential voltammetry (DPV)

The 10 mM dopamine solution was prepared by adding dopamine hydrochloride power (98%, Sigma-Aldrich) to 10 mL 0.01 M phosphate-buffered saline (pH 7.4, Sigma-Aldrich). This stock dopamine solution was added into 30 mL 0.01 M phosphate-buffered saline solution to get desired concentrations. Same Ag/AgCl reference and platinum counter electrodes as above were used in dopamine testing experiments, and all solutions were prepared with 18 MΩ·cm deionized water. A Gamry 1000E potentiostat was used for DPV tests, where the scan range was from −0.2 to 0.7 V, the step value was 2.0 mV, the sample period was 0.5 s, the pulse time was 0.05 s, and the pulse size was 25 mV. To calculate the LoD, a penalized spline version of the asymmetric least squares algorithm in Python (pybaselines.spline.pspline_asls) was used to subtract the baseline of DPV curves. To get a clear result, the smoothing parameter “lam” and penalizing weighting factor “p” were adjusted for each test. This option offers faster and automatic analysis for peak current calculation and improves the result of LoD calculation. Due to the difficulty of measuring the surface area of our electrode, the current was used here rather than current density.

#### Glucose detection by cyclic voltammetry (CV)

The 100 mM glucose solution was prepared by mixing ᴅ-(+)-glucose (99.5%, Sigma-Aldrich) in 10 mL 0.25 M NaOH (98%, Fisher Scientific) solution, and all solutions were prepared with 18 MΩ·cm deionized water. The stock glucose solution was added into 30 mL 0.25 M NaOH to get desired concentrations. Same reference and counter electrodes were used as above. The Gamry potentiostat was used for CV tests with scan range from −0.5 to 0.5 V, 30 mV/s, 1 mV step size (1 data point for 1 mV change, 30 data point per second), and four cycles. For noise reduction and statistical considerations, the mean value from the last three cycles were used for peak current calculation. The same Python algorithm was used for baseline subtraction.

## Data Availability

Data generated and analyzed during this study is available from the corresponding author upon reasonable request.
